# Innovative Culture and Firm Performance of Medical Device Companies: Mediating Effects of Investment in Education and Training

**DOI:** 10.3390/ijerph18178926

**Published:** 2021-08-25

**Authors:** Jeong-min Ryu, Sewon Park, Yoonseo Park, Jeongwon Park, Munjae Lee

**Affiliations:** 1 Department of Medical Device Management and Research, SAIHST, Sungkyunkwan University, Seoul 06355, Korea; jungmin94@skku.edu (J.-m.R.); se10919@g.skku.edu (S.P.); 2 Department of Bio Medical Engineering, Ajou University, Suwon 16499, Korea; yoonseo@ajou.ac.kr; 3 Department of Public Health Science, Graduate School, College of Health Science, Korea University, Seoul 02841, Korea; jw505@korea.ac.kr; 4 BK21 Four R&E Center for Learning Health Systems, Transdisciplinary Major in Learning Health Systems, Graduate School, Korea University, Seoul 02841, Korea; 5 Department of Medical Humanities and Social Medicine, Ajou University School of Medicine, Suwon 16499, Korea

**Keywords:** organization, innovative culture, education and training, job satisfaction, corporate performance

## Abstract

This research explored the mediating effect of investment in education and training relating to the innovative culture and organizational performance of medical device companies. We used the Human Capital Corporate Panel data provided by the Korea Vocational Competency Development Institute. In the industrial classification system of panel data, the industries related to medical devices were extracted and conclusively analyzed for 8629 workers and 368 companies. The independent and dependent variables were innovative culture and corporation performance, respectively. Investment in training and education was a mediating variable between the independent and dependent variables. Quantitative data were analyzed using SPSS software. A higher level of organizational satisfaction emerged in an innovative culture. Innovative culture positively affected organizational satisfaction. Further, investment in education and training to promote an innovative culture positively affected organizational satisfaction. Medical device companies should improve their performance by creating an innovative culture.

## 1. Introduction

The medical device industry in South Korea largely comprises small- and medium-sized companies (SMEs), which cannot exercise their self-sustaining power because they remain under political protection and have been negligent in their growth and development. The medical device industry belongs to small-scale multidisciplinary production—multidisciplinary and convergence technology ventures in medicine and engineering, high-tech industries in SMEs, high value-added industries in technology-intensive industries, and future industries in the 21st century [1,2]. According to the Food and Drug Administration’s report, in 2018, South Korea’s medical device market, based on production amount and import and export, was KRW 6.8179 trillion (approximately USD5.86 billion). This was a 10% increase from 2017, and the size of the domestic market grew at an average annual rate of 8% from 2014 to 2018. Considering the size of the domestic market, the number of companies is sizable. Only 90 companies produce over KRW 10 billion (approximately USD8.6 million) (3.3%), and 2300 small companies produce below KRW 1 billion (approximately USD900,000). Manufacturers with less than 20 employees account for more than 80% of the total [3,4]. Medical devices prioritize the safety and health of the human body while maintaining pace with technological development and innovative culture.

The medical device industry is experiencing a radical change of market scale and technology, based on enhanced living standards and an increased aged population. With an increase in the proportion of elderly patients owing to the rise in the retirement of baby boomers and life expectancy, demand for medical devices for chronic diseases is also expected to expand. The bio-health industry refers to manufacturing, medical and healthcare services, and medical devices. It is a promising new industry that has future growth potential and employment impacts. In the medical device industry, the first mover takes advantage of dominating the market and particularly has greater expectancy effects with active investment in new forms of technology. Major factors contributing to the growth of the medical device market include the aging trend, increased interest in health and improved well-being [5,6].

Advances in information technology, such as computers and the internet, in the late 20th century influenced the formation of a knowledge-based society. The key component of information technology is knowledge. The core aspect of the industry has been transforming from material-based to knowledge-based, and the significance of knowledge has progressed incomparably over time. Since extensive knowledge is shared and distributed, human resources have become a key element of corporate competitiveness: strong competitiveness of human resources enhances companies’ performance. Companies are actively investing in recruiting, educating, training and developing human resources. Education and training are essential activities for the advancement of both companies and their members.

Human resources refers to the knowledge, technological wealth or value that cannot be separated from people [7]. With the beginning of the fourth industrial revolution, technological change is accelerating, and the healthcare industry—based on information and communications technologies (ICT), such as mobile, the Internet of Things (IoT), big data and artificial intelligence (AI)—requires adaptation and response to rapid societal changes. Compared with the past, when human resources valued expertise, such as knowledge or skills required for a specific job, the ability to adapt to new environmental changes are more crucial. Beyond simple labor activities and physical capital, companies are investing in human resource development to foster improved performance. The ability to perform new tasks in the future is equally vital to increasing one’s productivity on current tasks. It is evident that education and training in organizations play a key role in enhancing the performance of the organization; however, relevant research focusing on medical device companies is limited.

In addition to the education and training of the members for human resource development, one of the factors that organizations must possess is the adoption of innovative change. The innovation culture within a company is known to improve internal and external productivity by facilitating rapid adaptation to changes in the external environment and integration within the organization. Diffusion of new ideas and knowledge activities in an innovative culture inspire members to operate more creatively. In particular, the innovation culture of the organization is essential for the sustainable development of SMEs. Innovation culture is an essential element of successful companies and serves as a source for SMEs to develop new technologies or products that are needed to pioneer new markets [8].

Existing studies related to the performance of medical device companies focus mainly on the research and development (R&D) of these companies, medical device technology commercialization and leader capacity of the companies. There is no comprehensive study on the relationship between organizational culture and organizational performance concerning the development of medical device companies. Therefore, we examined the mediating effects of investment in training and education relating to job satisfaction and the innovative culture of medical device companies. This study further seeks to articulate knowledge and ideas regarding the productive contribution of resources.

## 2. Literature Review

### 2.1. Organizational Culture

Organizational culture refers to beliefs, values, norms, customs, and so forth that are predominantly shared by members of an organization, and is an important factor affecting employees’ work-related behavior [9]. Organizational culture is a distinctive characteristic that makes one organization different from another. Organizational culture is formed through various factors such as the entrepreneur’s values, organizational history, sociocultural factors, business characteristics and the socialization process of the organization. Organizational culture is contained in the basic assumptions shared by members of the organization and affects the values that the organization stands for. These values are revealed in the corporate mission statement or the code of conduct [10]. They are expressed in the vision and mission statements of the organization and the way in which it operates and in the attitudes and actions of the employees. Organizational culture encourages members to maintain common goals and philosophies, induces collective immersion, increases the stability of the organizational system and performs the function of structuring the actions of the members. Alwi and colleagues [11] stated that workers’ performance can be improved through a healthy organization and that the more open culture is pursued, the more efficient organizational performance is shown. In addition, Nikpour [12] said that organizational culture directly affects employees’ job commitment, and organizational commitment affects organizational performance. Constructing a better organizational culture can improve organizational performance by strengthening the job commitment of workers. It is well established through several cases and empirical studies domestically and abroad that companies with a desirable and healthy organizational culture can produce better outcomes than their competitors. Companies with an outstanding organizational culture can influence profitability, growth rate and market performance, as well as innovation of new products and services by inducing consistent behavior from their members [13].

The concept of organizational culture was established in 1980; thereafter, extensive research has been conducted on organizational culture for more than 30 years, with over 4600 related papers published. Although prior studies on organizational culture have been undertaken, obtaining a single definition of the term is difficult: researchers define organizational culture in diverse ways. Herrison (1972) defined organizational culture as bureaucracy culture, power-oriented culture, matrix culture and atomized culture, while Deal and Kennedy (1982) defined it as a tough-guy culture, macho culture, work hard/play hard culture, bet-your-company culture and the process culture. Quinn (1983) defined the four cultural attributes that organizational culture can have and divided them into human resource development culture, open system culture, hierarchy culture and production-oriented culture. Since then, it has been developed and presented as a competing value framework and used as a tool for diagnosing organizational culture [14]. Among them, the validity and reliability of the competing value framework by Quinn and Rohrbaugh, which represents the organizational culture, has been verified by many scholars and is still widely applied [15].

**Figure 1:**
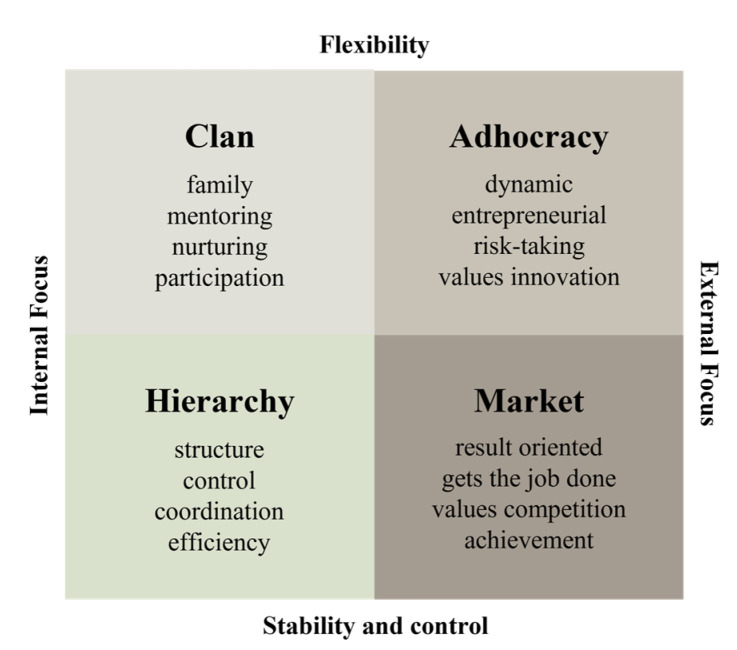
Competing value framework (Cameron & Quinn, 1999).

This study seeks to develop a discussion based on the competing value framework as a theoretical analysis of organizational culture. Quinn and Rohrbaugh’s competing value framework does not have the best criteria for evaluating the effectiveness of an organization. The core variables presented in various research models on organizational culture are combined to present an integrated analysis framework for organizational culture [16]. Unlike the early studies that addressed organizational culture qualitatively, the need for quantitative research has been emphasized, leading to the view that empirical research should be performed coherent to cultural characteristics. After Quinn and Rohrbaugh presented the competing value framework, Quinn and Kimberly (1984) applied the competing value framework to the organizational culture for the first time. Moreover, Cameron and Quinn (1999) developed the organizational culture assessment instrument, an organizational culture evaluation tool, and applied the competing value framework to the diagnosis and management of organizational culture [17]. Therefore, this study utilized Cameron and Quinn’s organizational culture competing value framework: clan culture, adhocracy culture, hierarchy culture and market culture (Figure 1).

Adhocracy culture focuses on adaptability to the internal environment of the organization, and emphasizes the flexibility of the organization and the creativity of the organization members. The paradigm of the economy is rapidly changing owing to the fourth industrial revolution, and companies are trying to adapt to the environment by changing their organizational culture. Medical device companies closely related to ICT and IoT emphasize flexibility and creativity to cope with real-time changing technologies. Medical device companies should develop medical devices that incorporate new technologies and check changes in stakeholders and government policies to provide them to the market. ICT companies need to grasp technological changes and market trends in real-time; thus, organizational change and innovation and values such as creativity and a challenging spirit should be a top priority. In recent studies, innovative culture emphasizes originality and acceptance of ideas in decision-making. To maintain an innovative culture, organizations are encouraging employees to regularly change and to demonstrate creativity through strengthening their capabilities. Medical device companies are expected to form an innovative organizational culture that encourages creativity through strengthening the capacity of employees to develop new medical devices and adapt to changing environments [18]. In this study, adhocracy culture is defined as an innovative culture.

### 2.2. Innovative Culture

Innovation is an activity that creates new value and, to this end, it executes creative thinking within the organization. Innovation is the most efficient way to achieve competitive advantage and sustainable development in the market, and innovative companies are trying to develop their ability to adapt to the market. The creativity of an organization leads to innovation within the company, and the constant learning of the organization affects the growth of the company. When an organizational culture or environment promotes innovation capacity and supports personal growth and development, the organizational culture can be classified as an “innovation culture”. Companies are creating better competitive advantages by utilizing innovation to realize sustainable development. Innovative culture involves using innovation as an important strategic tool in competition by developing internal and external situations such as business improvement and productivity improvement [19]. In an innovation-oriented society, competitive environment, creativity, innovative attempts and risk-taking actions are promoted. To accomplish this, numerous attempts and free speech practices are highlighted by the members [20]. Innovation-oriented assets, such as innovative culture and employee knowledge diversity, have been suggested as vital inputs for the development of new products or services [21].

Employees can share their values and beliefs concerning the development and pursuit of novel business ideas in an innovative culture, which is of central significance. Recently, companies are paying attention to open innovation to accelerate the innovation culture. Open innovation refers to accepting external knowledge and bringing about innovation in the company. The more employees share various knowledge, the more they can create new value in combination with external knowledge [22].

An innovative culture is defined by the delegation of decision-making powers for employees to pursue their own ideas [23]. Organizations that lack the required culture would find knowledge sharing to be limited and challenging, since organizations comprise employees who possess the requisite knowledge for the organization to learn and develop [24]. In addition, companies with an innovative culture are highly likely to be internally oriented and exceedingly competitive since they are expected to successfully embrace novel ideas and processes [18].

Innovative culture has a positive impact on organization innovation. Furthermore, innovative culture encourages the autonomous innovation activities of individuals; groups and organizations require a province to support innovative activities in the field and need innovative thinking and action from employees [25]. Aggarwal and colleagues [26] said that creating an innovative culture and giving structural authority to members would create a harmonious environment within the organization, which would improve job satisfaction and organizational commitment. Therefore, if an innovation culture is created, it will foster employees’ organizational commitment and positively affect organizational performance.

In an innovative culture, the flexibility and discretion of the members are also important. To cope with environmental changes, we will develop new products or services for survival of correspondence to the external environment through strengthening the capacity of organizational members. In an innovative culture, the organizational change to cope with the new market is the greatest value. Through strategies such as organizational performance, flexibility and human resource development, organizational innovation should be strengthened to create a culture that can secure a competitive advantage [27]. Medical device companies emphasize the flexibility and creativity of the organization to develop new medical technologies. In addition, to strengthen the capacity of the company, an innovation culture through human resource development such as job development is necessary. Therefore, to analyze the effect of innovation culture on medical device companies’ corporate performance, the following hypothesis was established:

**Hypothesis** **1.** *Innovative culture will influence organizational performance.*

### 2.3. Education and Training

Education and training have been recognized as the competitiveness factor of the organization. In particular, to lead innovation, education and training are drivers of change for discovering future growth engines and R&D [28,29]. Since education and training have been highlighted as a way to improve the core competencies of employees, governments and local authorities are also implementing education and training that can effectively sustain them.

According to a survey on the Supply and Demand of Industrial Technology Personnel (2019) [30], investment in education and training has been declining, particularly in SMEs. Training participation as well as expenses and time allotted in South Korea compared with the European Union (EU), are conspicuously low, especially in small companies (Table 1). The inferior performance of technology innovation in SMEs further contributed to the problem of development of workforce skills, and the actual rate of training practice was low owing to employer awareness, technology development (automation), simplified tasks and workforce problems (temporary, foreigners). Companies are inclined toward education and training and are promoting investment in the development of human resources. Clan culture companies emphasize mutual trust among members and tend to respond passively to environmental changes, which leads to more investment in emphasizing collective consciousness rather than education and training. Companies that have a hierarchical culture are likely not to actively invest in education and training because they focus on maintaining the status quo of the organization. Companies prioritize achieving corporate goals and emphasize efficient organizational management. In the market culture, they focus on corporate activities in the external environment; thus, they are expected to invest in external factors such as market change and the latest technology rather than education and training. In contrast, adhocracy culture focuses on securing resources for growth to respond creatively and quickly to changes in the market environment. To overcome the rapidly changing environment and competition, innovative culture companies will accumulate more knowledge and actively conduct education and training [31]. Therefore, companies pursuing an innovative culture are likely to conduct active education and training as they require relatively more knowledge.

In the face of global competition, many companies are endeavoring to ensure sustainability and generate effective results. The evolution of economic activity in a knowledge economy demonstrates the value of knowledge or intellectual assets as key production factors in the survival and performance of a company. Acquisition of new skills and education have become key functions. Nurturing experienced employees is an assured way to achieve an edge over other companies. Similarly, developing human resources entails enhancement of knowledge, skills and abilities of employees with the aim of improving performance within the organization. It relates to reorganizing the organizational structure and encouraging the system to promote job performance and motivating members for self-improvement. Through education and training, members of the organization can develop performance skills and address problems or deficiencies in their work performance [32].

Unlike in the past, knowledge and skills of an employee have now become a significant factor influencing the competitiveness and performance of the company, and human resources have become an important source of competitive advantage. Consequently, companies are emphasizing commitment and investment in developing human resources, and enormous amounts are invested in education and training worldwide [33]. Companies are committed to the continuous renewal of their competitive advantage through consistent innovation and the advancement of new knowledge and capabilities. In addition, companies’ ability to consistently innovate their knowledge assets—as a diverse capability—is essential for potential success.

Education and training refer to the systematic acquisition and development of the requisite knowledge, technology, and attitudes to appropriately perform tasks or to enhance employees’ performance. Through diverse types of education and training, the knowledge of employees can be enriched, and essential information can be actively shared between the members. Further, an environment in which members can recognize opportunities for self-development in the future can be cultivated. The productivity of human resources influences corporate performance. Human resources in an organization are acknowledged as a source of competitive advantage; hence, the development and management of intangible resources is growing steadily—not only at the corporate level, but also at the individual and national level. This is because the development of human resources is expected to accomplish considerable improvement in the knowledge, ability, skills, and organizational performance through the education and training of the members [34].

Human resources are extremely intricately connected to a country’s economic and social growth at a macro level. The higher the level of economic and social culture, the higher the level of human resources, such as managers and technical workers, and the greater the degree to which human resources, compared with funds and material resources, contribute to organizational performance. Therefore, the higher the economic and cultural level, the greater the value of successful human resource management, such as securing and developing high-quality human capital [35]. As such, human resources are not only an essential asset of an organization, they are also a strategic resource that plays a key role in an organization’s performance and growth [36].

Some claim that investment in education and training does not directly affect organizational performance [37]. As such, diverse perspectives on corporate education preparation and performance enhancement have been raised and debated continuously. Identifying the relationship between education and training and improving performance would provide an essential basis for businesses to determine their investment in education and training, which is considered necessary.

Companies that pursue innovative culture intend to create an organizational culture that can lead to innovative and creative ideas through investment in education and training. Innovative cultural companies will invest more in education and training as they continue to engage in processes that enable their employees to generate the best value through educational programs on new technologies and roles. Investment in education and training is focused on changing the internal organization in a flexible and innovative direction, and thus enhancing management performance [38]. Hence, we proposed the following hypothesis:

**Hypothesis** **2.** *Education and training investment will influence organizational performance.*

### 2.4. Organizational Performance

Improving employee performance is essential for an organization to achieve a competitive advantage. However, to accomplish this objective, an organization should ensure that its employees are satisfied with their work.

Kulachai and colleagues [39] have confirmed that there is a positive relationship between job satisfaction and employee performance. Organizational performance does not mean only financial performance, it also represents individual performance that includes job satisfaction, quality of work life and self-growth [40,41]. Financial management performance is measured primarily based on revenue, net profit and so forth, using quantified indicators. Non-financial management performance reflects the intangible value of the company and is measured based on satisfaction, immersion and labor productivity. Since financial performance is used as a concept related to short-term performance and non-financial management performance is measured by diverse long-term criteria, limitations of financial performance can be addressed. Medical device products take a long time to produce significant results after sales and are determined by external factors, such as government policies, in addition to product competition; hence, non-financial performance was used as an indicator of success [42].

Without enhancing individual performance, it is challenging to improve the performance of an organization and is not desirable for an organization to persist. Therefore, the link between individual and organizational performance improvement must always be well-harmonized. These organizational achievements are described in a variety of ways, and the following are examples from various scholars [43].

Some believe it is simply how content individuals are with their respective jobs, whether they appreciate the job or its individual aspects or facets, such as the nature of work or supervision. Locke [44] describes job satisfaction as “the pleasurable emotional state resulting from the appraisal of one’s job as achieving or facilitating the achievement of one’s job values.” De Nobile [45] defined job satisfaction as “the extent to which a staff member has favorable or positive feelings about work or the work environment.” Davis and colleagues [46] described job satisfaction as “a set of favorable or unfavorable feelings for the employees to perceive their work and that determine the possibility of a major disposition to achieve higher performance.” In addition, job satisfaction is a pleasant and optimistic feeling that organizational employees experience after assessing their work and work experience [47].

Organizational satisfaction was argued as a predictor of a company’s potential success, and the results demonstrated that the organization’s satisfaction was closely linked to employee absenteeism and productivity. Byun and Kim [48] also argued that organizational satisfaction is meaningful as a proxy index for the evaluation of organizational performance. Job satisfaction is a pleasurable emotional state that results from a sense of achievement in the workplace [49]. In addition to being identified as a positive feeling that emerges from cherishing one’s job and work-related experiences, job satisfaction has also been described as the feeling evoked in response to a mental comparison of expected and actual job outcomes [50,51].

In short, job satisfaction is the degree to which individuals like their jobs [52]. Mutual respect is also essential for fostering healthy workplace relationships. When thoughts, ideas and feedback are respected, it cultivates a collaborative atmosphere that encourages individuals to acquiesce and work toward the same objectives. This is where diversity takes effect. When the diversity in opinions is embraced and encouraged, everyone feels respected, which results in stronger relationships at work.

Companies that pursue innovative culture intend to create an organizational culture that can create innovative and creative ideas through investment in education and training. Innovative cultural corporates will invest more in education and training as they continue to engage in processes that enable their employees to generate the best value through educational programs on new technologies and roles. Investment in education and training is focused on changing the internal organization in a flexible and innovative direction. Through this, it enhances management performance [38]; hence, we established the following hypothesis:

**Hypothesis** **3.** *A company’s education and training investment will mediate the relationship between organizational culture and organizational performance.*

## 3. Materials and Methods

### 3.1. Research Model

**Figure 2:**
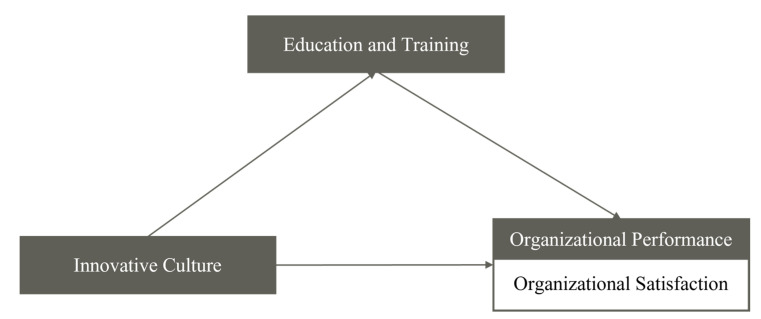
Research model.

Based on the theoretical background discussed above, we sought to grasp the mediating effect of education and training investment relating to innovative culture and organizational performance of medical device companies. For this purpose, innovative culture, organizational performance and organizational satisfaction were established as the independent variable, dependent variable and sub-concept, respectively. In addition, investment in education and training was established as the mediating variable between the independent and dependent variables. The research model is illustrated in Figure 2.

### 3.2. Data Collection

The current study used the Human Capital Corporate Panel (HCCP) data provided by the Korea Vocational Competency Development Institute. Beginning with the first survey in 2005, the HCCP Survey has been conducted every two years and now presents the seventh survey results. The research on the HCCP data can identify the quantitative and qualitative level of South Korean corporate workforce, and it can grasp the process and contents of accumulating human resources in a company. HCCP data are accumulated by companies and workers, and the questionnaire consists of items on general management, human resource management, workforce status, human resources development and R&D. The survey of workers consists of participation, the effects of education and training, corporate culture and job satisfaction.

Data from the fifth (2013) to the seventh (2017) years were used to analyze the mediating effects of investment in education and training relating to innovative culture and organizational performance of medical device companies. Data samples were provided to industries relevant to medical devices extracted from the industry classification system for panel data (Table 2). Recently, as AI-based medical devices are being actively developed, medical device companies are not limited to manufacturing and include AI-related video and information services fields. Data from 8629 employees from 368 companies were analyzed, excluding those with no or partial responses to the questionnaire.

### 3.3. Description of Variables

Organizational performance was measured with the organizational satisfaction provided by HCCP data, which utilizes a 5-point Likert scale. In addition, innovative culture was measured with the response to the innovation-oriented organizational culture among HCCP data, which also utilizes a 5-point Likert scale. The mediating variable, education and training investment, was measured as the total amount of investment in education and training, which was converted to logarithmic values.

### 3.4. Statistical Analysis

Results were analyzed with SPSS 25.0 (IBM, Armonk, NY, USA). First, a correlation analysis was conducted to determine the relationship between each variable. Second, a reliability analysis was conducted to verify the validity and reliability of the variables. Third, a regression analysis was performed to examine the causal relationship between innovative culture and corporate performance and analyze the mediating effect of investment in education and training.

## 4. Results

### 4.1. Correlation Analysis

To examine the relationship between variables, a correlation analysis was conducted, and the results are indicated in Table 3. Innovation culture and organizational satisfaction level were positively correlated (r = 0.67).

For the validity and reliability verification of variables, exploratory factor and reliability analyses were conducted; main component analysis was used as a factor extraction method, and varimax rotation was used (Table 4). The Kaiser-Meyer-Olkin (KMO) value was 0.735, and the significance probability of Bartlett’s χ^2^ value was less than 0.05, which was judged to be appropriate for the factor analysis model. The cumulative proportion was 88.67%, which was judged to be high in the explanatory power of the two components, and all the factor loadings exceeded 0.4, satisfying the validity of the measurement variables. Cronbach’s α was 0.857; thus, the questionnaire was deemed to have good reliability.

### 4.2. Regression Analysis

A regression analysis was conducted to verify the effectiveness of education and training investment relating to the effect of innovative culture on organizational satisfaction (Table 5).

Model 1 represents the relationship between the dependent variable, which is organizational satisfaction and innovative culture, and Model 2 indicates the relationship between investment in education and training and organizational satisfaction. Model 3 indicates the relationship between innovative culture, investment in education and training and organizational satisfaction.

The R^2^ value increased to 12.8% in Model 1, 15.1% in Model 2, and 15.6% in Model 3, and the interaction of innovative culture and educational training investment was significant. Investment in education and training mediated the relationship between innovation culture and organizational satisfaction.

As illustrated in Model 1, innovative culture positively affected organizational satisfaction. As presented in Model 3, investment in education and training to promote an innovative culture positively affected organizational satisfaction.

## 5. Discussion and Conclusions

This study identified how innovation culture influences corporate performance, notably the satisfaction of employees, relating to investment in education and training. First, the innovative culture of medical device companies had a positive effect on organizational satisfaction. This is consistent with studies that indicated a higher level of job satisfaction among employees of corporates with innovative cultures [53]. Companies that have an innovative culture create an environment in which they can generate new ideas by positively accepting the creative thinking of workers. This seems to improve the organizational satisfaction of employees by enabling them to demonstrate their talents and expertise in job performance [54]. Workers of medical device companies have expertise in each individual work area, such as new technology, government policies, regulatory affairs, clinical trials and marketing, and they must create solutions based on their expertise in the problems they recognize while performing their work [1]. The more a medical device company has an innovation culture, the higher the organizational satisfaction of its workers because it must respond actively according to the individual’s job performance.

Second, investment in education and training of medical device companies positively affected organizational performance. This is similar to the findings of a study that showed that a trained workforce is needed to maintain a competitive advantage in the global economy [55]. Investment in education and training is an effective way to enhance the profitability of companies by encouraging the growth of companies and the development of employees’ capabilities. Investment in education and training should be introduced for the future growth of companies. The structure of the medical device industry is rapidly changing owing to external factors such as the fourth industrial revolution and COVID-19. For a company’s business activities to be performed smoothly, it is necessary to cultivate expertise to cope with the rapidly changing market. Through education and training for workers, companies can strengthen workers’ expertise and revitalize the organization, thereby improving organizational performance. In addition, the medical device industry is one of the industries that the government is fostering as a future food industry. For the development of the medical device industry and the survival of medical device companies, the government is promoting “A Specialized Graduate Program of Medical Industry” to nurture medical device specialists. With the expansion of specialized graduate school projects in the future, it will be possible to improve the viability and performance of medical device companies by fostering human resources.

Third, the organizational culture of medical device companies had a positive effect on organizational performance by mediating the association of investment in education and training. This differs from previous results that showed that the more companies form a clan culture, the higher the organizational performance through education and training as compared with companies that employ an innovative culture [54].

As a result of the study, it was found that the investment in education and training of medical device companies plays a mediating role between the company’s innovative culture and organizational performance. This indicates that continuous investment in education and training is necessary to create an innovative culture and to satisfy workers’ job satisfaction to improve the organizational performance of medical device companies. As the medical device industry needs to be sensitive to diverse fields and ever-changing technologies, businesses must be able to respond flexibly to a rapidly changing environment. Government regulations, ethical values and cost effectiveness must also be considered as the value of multi-stakeholder conflicts. In response, medical device companies are fostering an innovative culture that emphasizes flexibility and outward-facing change. In addition, it is an important factor in the development of new products to secure a competitive advantage in the industry, and for employees to embrace innovative values and produce new products, investment in education and training can improve the organizational satisfaction of workers and increase organizational performance.

## 6. Limitations

This study was intended to suggest a strategic direction for the rapidly growing medical device industry, but it has the following limitations. It did not solve the problem of endogeneity between variables. Most of the studies that empirically analyze causality, such as this study, test the hypothesis by assuming a unidirectional effect with the dependent variable and the independent variable. However, endogeneity can occur if variables are determined interdependently. Ullah et al. (2017) said that regression analysis can cause endogeneity problems and lead to incorrect interpretation of the results. Additionally, in general, various methods such as two-stage least squares (2SLS), fixed-effects models, generalized method of moments (GMM) and dynamic panel GMM are used to solve the endogeneity problem [56]. Therefore, in future studies, it is necessary to increase the accuracy of the analysis results by controlling the endogeneity between variables. In addition, it is necessary to overcome the limitations of the utilization of panel data. In this study, there was a limitation in the selection of variables due to the use of panel data, and it is necessary to additionally include variables such as company size, CEO characteristics and presence or absence of incentives. Therefore, in future research, it is necessary to solve the endogeneity problem and measure the variables in depth by utilizing various variables that are affected within the same industrial structure of the medical device industry.

## Figures and Tables

**Table 1:** Current investment status in education and training in the EU and South Korea.

	Training Participation Rate		Training and Education Expenses/Labor Costs		Training Hours per Employee	
	South Korea	EU	South Korea	EU	South Korea	EU
Total	34.1	40.8	0.5	1.7	6.5	12.1
Small (10–29 people)	22.8	30	0.09	1.3	4	11.9
Small (30–99 people)	24.4		0.2		3.8	
Medium (100–299 people)	40	37.2	0.3	1.6	5.9	11.7
Large (≥300 people)	47.5	47.7	0.75	2	11.8	12.3

EU = European Union.

**Table 2:** Classification of medical device companies.

KSCI	Field Name
	Manufacturing
26	Electronic parts, computer, video, acoustics and telecommunication equipment manufacturing
27	Medical, precision, optical equipment and clock manufacturing
	Publishing, video, broadcasting and telecommunications and information services
61	Communication
62	Computer programming and system integration and management
63	Information service industry

KSCI = Korean Standard Industrial Classification.

**Table 3:** Correlation analysis results.

Variables	Mean	Standard Deviation	Correlation Rate	
			(1)	(2)
(1) Innovative culture	2.47	0.56	1	
(2) Organizational satisfaction	3.20	0.80	0.67 **	1

** *p* < 0.05.

**Table 4:** Validation of validity and reliability.

Variables		Communality	Component	
			1	2
Innovative culture	Innovative 1 Innovative 2 Innovative 3	0.821 0.895 0.898	0.825 0.864 0.857	
Organizational satisfaction	Satisfaction 1	0.465		0.798
Cronbach’s α			0.857	
Eigenvalue			1.79	1.68
Explain dispersion (%)			44.72	41.95
Cumulative dispersion (%)			44.72	86.67
KMO = 0.735, Bartlett’s χ^2^ = 6434.088, *p* < 0.001				

**Table 5:** Regression analysis results.

Variable	Dependent Variable: Organizational Satisfaction		
	Model 1	Model 2	Model 3
(Constant)	5.82 (1.04)	5.42 (1.03)	8.14 (2.67)
Innovative culture	0.53 *** (0.13)	0.52 *** (0.12)	0.19 *** (0.32)
Investment in education and training		0.11 ** (0.05)	−0.51 ** (0.56)
Innovative culture * Investment in education and training			0.08 * (0.07)
R^2^	0.12	0.15	0.16
F	17.58 ***	11.80 ***	8.29 ***

* *p* < 0.1, ** *p* < 0.05, *** *p* < 0.001.

## Data Availability

Data was obtained from Korea Research Institute for Vocational Education and Training, KRIVET and are available at https://www.krivet.re.kr/ku/ha/kuCDADs.jsp (accessed on 28 July 2021) with the permission of KRIVET.
